# Association of the type of counselor with suicidal ideation and suicide attempts: a survey of South Korean adolescents

**DOI:** 10.1186/s13034-021-00414-1

**Published:** 2021-11-06

**Authors:** Yunkyung Kim, Wonjeong Jeong, Jieun Yang, Sang Ah Lee, Eun-Cheol Park

**Affiliations:** 1 Department of Public Health, Graduate School, Yonsei University, Seoul, Republic of Korea; 2 Institute of Health Services Research, Yonsei University, Seoul, Republic of Korea; 3 Department of Preventive Medicine, Yonsei University College of Medicine, 50 Yonsei-ro, Seodaemun-gu, Seoul, 03722 Republic of Korea

**Keywords:** Suicidal ideation, Suicide attempt, Counselor, Adolescent, South Korea

## Abstract

**Background:**

Counseling for adolescents who consider or attempt suicide may help reduce suicide rates. However, the impact of the type of counselor (e.g., father, mother, sibling, friend, teacher, other) on suicidal ideation/suicide attempts remains unclear. Therefore, we examined this association in Korean adolescents.

**Methods:**

Using data from the 2015 Korea Youth Risk Behavior Web-based Survey of 65,485 adolescents, we examined risk factors for suicidal ideation/suicide attempts according to sex using a multiple logistic regression analysis. In a subgroup analysis, we investigated the associations between counselor type and suicidal ideation/suicide attempts stratified by the cause of stress for both sexes.

**Results:**

Male participants were less likely to consider suicide when counseled by their mother (odds ratio [OR] 0.80; 95% confidence interval [CI] 0.70–0.90) or friend (OR 0.89; 95% CI 0.80–0.99) and to attempt suicide when counseled by a friend (OR 0.74; 95% CI 0.60–0.92) than were those who did not receive any counseling. Female participants were less likely to attempt suicide when counseled by their mother (OR 0.66; 95% CI 0.53–0.82) or friends (OR 0.70; 95% CI 0.58–0.83) than when not counseled. Furthermore, adolescents with achievement/career choice-related and family problems were at a lower risk of suicide ideation/suicide attempts when counseled by their mother or friend than when they received no counseling.

**Conclusions:**

Suicidal ideation/suicide attempts showed different associations for both sexes in accordance with the type of counselor. Even if counseling by specialists for issues such as achievement/career choice-related or family matters is needed, the risk of suicide could be reduced through counseling by the people around them. Therefore, adolescents should form mutually supportive relationships through active communication with surrounding people.

## Background

Since 2003, South Korea has had the highest suicide rate per 100,000 people among all member countries of the Organization for Economic Co-operation and Development (OECD); in 2016, 13,092 people (1.5/h) committed suicide [1]. Suicide is the leading cause of death in teenagers and young adults in South Korea, accounting for 30.0% of deaths among those in their 10 s, 43.8% among those in their 20 s, and 35.8% among those in their 30 s [2]. Moreover, it has been reported that substantial sex-associated differences exist in suicidal behaviors among adolescents [3, 4]. The risk factors associated with suicidal behavior are also known to differ between male and female adolescents, as psychiatric problems which affect suicide risk may vary in male and female adolescents [3, 5]. Suicide is, therefore, a serious problem for young people in South Korea. While the suicide rate is important to track, suicidal ideation/suicide attempts, which comprise pre-suicide stages, are more meaningful. Adolescents who think about or attempt suicide could be considered a key latent suicide risk group [6]. Preventing suicide in adolescents is, therefore, important because it may help reduce actual suicide related deaths in South Korea [7].

Many studies conducted worldwide have documented the positive effect of counseling on suicide [8, 9]. That said, the proportion of Korean adolescents who resolve their problems by themselves has risen from 16.9% in 2010 to 21.8% in 2016 [10]. One explanation for this is the widespread use of smartphones and improved internet accessibility, thus enabling adolescents to easily find the information they need to deal with their problems independently [11]. We know that the average size of households in Korea has reduced from 5.3 in 1970 to 2.5 in 2016 [11], indicating that the family structure in South Korea has gradually nuclearized. A decrease in the number of family members (parents/siblings) could mean diminished interactions with those whom individuals are most likely to be psychologically dependent upon. Moreover, according to the 2018 survey results of Child Fund Korea, a total of 571 respondents only spent an average of 13 min a day (on weekdays) with their family [12]. Therefore, we examined the relationship between the existence/types of counselors and suicide in adolescents; thus, this study has implications for suicide prevention.

Although committing suicide and attempted suicide are complex behaviors, they are known to be highly associated with internalizing psychopathology (e.g., depression and anxiety), demographic characteristics (e.g., age), and social factors (e.g., stressful life events) [13, 14]. It has been reported that patients with a history of major depression were more likely to attempt suicide compared to healthy controls [15]. Moreover, stress has been identified as a key determinant of suicidal behavior [13]. Previous studies have shown that the level of perceived stress due to various reasons, such as conflict with family or academic problems, is highly associated with suicidal behaviors [16]. Therefore, in addition to mental health response, a social and public health response is crucial in preventing suicidal behaviors in troubled adolescents [17].

Furthermore, it is necessary to investigate the association of the type of counselor with suicidal ideation and suicide attempts in South Korean adolescents after adjusting for other demographic, socioeconomic, and health-related characteristics. It is practically impossible for all youths who think about or attempt suicide to have access to professionally trained counselors [18]. Given this reality, the purpose of this study was to examine whether suicidal ideation or suicide attempts of an adolescent are associated with a counselor who was in close proximity with the adolescent.

## Methods

### Study population

The data used in this study were taken from the 2015 Korea Youth Risk Behavior Web-based Survey (KYRBWS) conducted by the Ministry of Education, Ministry of Health and Welfare, and Korea Centers for Disease Control and Prevention (KCDC). The KYRBWS is an annual anonymous online self-reported survey to investigate Korean adolescent health-related behavior. The data collected is used to conduct cross-sectional studies. The KYRBWS was approved by the KCDC Institutional Review Board (2014-06EXP-02-P-A) in 2014. Since 2015, the ethics approval for the KYRBWS was waived by the KCDC Institutional Review Board under the Bioethics and Safety Act and opened to the public. All participants provided informed consent to participate in the KYRBWS and were guaranteed anonymity. The KYRBWS is an anonymous, Internet-based, self-administered structured questionnaire that uses a complex research design, including multistage sampling, stratification, and clustering [19]. The survey comprises 123 questions assessing 15 health-related behaviors. The target population of the survey are students in grades 7 through 12 in South Korea. For each grade level, sample classes are chosen at random from schools across the country, with all students in the selected classes being chosen as the sample students [20]. Given that questions concerning the causes of stress were only included in 2015, we selected this year as the study period. We excluded data from 2558 individuals because of missing responses, and the response rate was 96.7%. As only individuals who had concerns needed a counselor, which was our main interest, we did not include those who reported no stress. Finally, the survey was completed by 65,485 adolescents (male, n = 33,254, 50.78%; female, n = 32,231, 49.21%), and they were included in the study.

### Variables

The variable of interest was the type of counselor, which was assessed for each adolescent by asking, “Who do you usually seek counsel from when you have a problem?”. The answers were classified into one of the following seven categories: (1) father, (2) mother, (3) sibling, (4) friend, (5) teacher, (6) other, and (7) none.

The dependent variables were suicide ideation and suicide attempts, which were assessed by the two following KYRBWS questions: “Have you seriously considered suicide in the past 12 months?”, and “Have you attempted suicide in the past 12 months?”. The responses to both questions were either “yes” or “no.”

The covariates were sex, middle/high school, having parents (“father,” “step-father,” “mother,” and “step-mother” responses were re-coded into “both or single-parent family,” and “none”), perceived household economic status (“high,” “middle-high,” “middle,” “middle-low,” and “low” responses were re-coded into “high,” “middle,” and “low”), academic achievement (“high,” “middle-high,” “middle,” “middle-low,” and “low” responses were re-coded into “high,” “middle,” and “low”), experience of alcohol/smoking (at least a glass of alcohol/a cigarette, responses to which were yes/no), subjective happiness (“very happy,” “happy,” “neither happy nor unhappy,” “unhappy,” and “very unhappy” responses were re-coded into “happy,” “neutral,” “unhappy”), stress awareness (“very high,” “high,” “low,” and “very low” responses were re-coded into “high” and “low”), depression (ever having experienced a sense of sadness or despair to an extent that daily life was disrupted in the past 12 months, responses to which were yes/no), and sleeping time for fatigue recovery (“very sufficient,” “sufficient,” “normal,” “not sufficient,” and “not at all” responses were re-coded into “sufficient,” “normal,” and “not sufficient”). We regrouped the responses for causes of stress according to the frequency of each category, as follows: “achievement/career choice,” “family problems,” “friendship relations,” “appearance” and “others.” Individuals who answered “conflict with parents” and “family environment” were classified as having “family problems.” Categories with less than 1% responses were classified as others. The analysis was conducted according to sex because biological and psychological characteristics differ by sex.

### Statistical analysis

Chi-square tests were used to analyze the general characteristics of the study population. A multiple logistic regression analysis was performed to examine the association between suicidal ideation/suicide attempts and other variables. Subgroup analyses were also performed using multiple logistic regression to examine the associations between types of counselors and suicidal ideation/suicide attempts stratified by causes of stress for both sexes. The results were reported as odds ratios (ORs) and 95% confidence intervals (CIs). Statistical significance was defined as P < 0.05. All data management and statistical analyses were conducted using SAS software (version 9.4; SAS Inc., Cary, NC, USA).

## Results

Table 1 shows the general characteristics of the study population by sex, which included 65,485 participants (33,254 males, 32,231 females). Of these, 9.7% (n = 3242) of male participants and 14.0% (n = 4526) of female participants experienced suicidal ideation, while 1.9% (n = 628) of male participants and 3.0% (n = 972) of female participants had attempted suicide. Among the male participants, 30.1% (n = 10,013) had no counselors, 28.2% (n = 9389) were counseled by a friend, 24.5% (n = 8131) by their mother, and 2.6% (n = 858) by their teacher. Meanwhile, among female participants, 44.2% (n = 14,254) were counseled by a friend, 29.7% (n = 9583) by their mother, 13.4% (n = 4306) had no counselor, and 1.1% (n = 367) were counseled by their teachers.

**Table 1:** General characteristics of the study population

Variables	Total N = 33,254	Male participants				Total N = 32,231	Female participants			
		Suicidal ideation	P-value	Suicide attempt	P-value		Suicidal ideation	P-value	Suicide attempt	P-value
		Yes		Yes			Yes		Yes	
	N (%)	N (%)		N (%)		N (%)	N (%)		N (%)	
Type of counselor			< 0.0001		< 0.0001			< 0.0001		< 0.0001
Father	2486 (7.5)	208 (6.7)		48 (1.5)		621 (1.9)	94 (3.0)		30 (1.0)	
Mother	8131 (24.5)	503 (2.8)		92 (0.5)		9583 (29.7)	886 (5.0)		173 (1.0)	
Sibling	1405 (4.2)	136 (3.6)		31 (0.8)		2390 (7.4)	254 (6.7)		56 (1.5)	
Friend	9389 (28.2)	974 (4.1)		167 (0.7)		14,254 (44.2)	2010 (8.5)		401 (1.7)	
Teacher	858 (2.6)	119 (9.7)		26 (2.1)		367 (1.1)	89 (7.3)		25 (2.0)	
Other	972 (2.9)	132 (7.8)		32 (1.9)		710 (2.2)	130 (7.7)		35 (2.1)	
None	10,013 (30.1)	1170 (8.2)		232 (1.6)		4306 (13.4)	1063 (7.4)		252 (1.8)	
Middle and high school			0.0278		0.1161			0.0009		< 0.0001
Middle school	16,627 (50.0)	1561 (4.8)		334 (1.0)		16,013 (49.7)	2353 (7.2)		593 (1.8)	
High school	16,627 (50.0)	1681 (5.1)		294 (0.9)		16,218 (50.3)	2173 (6.6)		379 (1.2)	
Having parents			< 0.0001		< 0.0001			< 0.0001		< 0.0001
Both or single parents	32,809 (98.7)	3141 (4.8)		587 (0.9)		32,011 (99.3)	4469 (6.9)		942 (1.5)	
None	445 (1.3)	101 (15.2)		41 (6.2)		220 (0.7)	57 (8.6)		30 (4.5)	
Perceived household economic status			< 0.0001		< 0.0001			< 0.0001		< 0.0001
High	12,510 (37.6)	1150 (5.0)		232 (1.0)		10,641 (33.0)	1327 (5.7)		303 (1.3)	
Middle	15,107 (45.4)	1266 (4.1)		220 (0.7)		15,945 (49.5)	1948 (6.3)		370 (1.2)	
Low	5637 (17.0)	826 (7.3)		176 (1.6)		5645 (17.5)	1251 (11.1)		299 (2.7)	
Academic achievement			< 0.0001		< 0.0001			< 0.0001		< 0.0001
High	12,514 (37.6)	1107 (4.5)		197 (0.8)		12,023 (37.3)	1470 (6.0)		281 (1.1)	
Middle	8984 (27.0)	795 (4.3)		145 (0.8)		9323 (28.9)	1138 (6.2)		229 (1.3)	
Low	11,756 (35.4)	1340 (5.9)		286 (1.3)		10,885 (33.8)	1918 (8.5)		462 (2.0)	
Alcohol			< 0.0001		< 0.0001			< 0.0001		< 0.0001
Yes	6555 (19.7)	921 (8.6)		212 (2.0)		4142 (12.9)	913 (8.5)		241 (2.3)	
No	26,699 (80.3)	2321 (4.2)		416 (0.8)		28,089 (87.2)	3613 (6.6)		731 (1.3)	
Smoking			< 0.0001		< 0.0001			< 0.0001		< 0.0001
Yes	3929 (11.8)	605 (12.3)		179 (3.6)		1004 (3.1)	330 (6.7)		128 (2.6)	
No	29,325 (88.2)	2637 (4.4)		449 (0.7)		31,227 (96.9)	4196 (6.9)		844 (1.4)	
Subjective happiness			< 0.0001		< 0.0001			< 0.0001		< 0.0001
Happy	22,143 (66.6)	1156 (2.7)		231 (0.5)		20,191 (62.6)	1484 (3.5)		304 (0.7)	
Neutral	8474 (25.5)	1069 (6.0)		200 (1.1)		9350 (29.0)	1723 (9.7)		322 (1.8)	
Unhappy	2637 (7.9)	1017 (19.1)		197 (3.7)		2690 (8.4)	1319 (24.8)		346 (6.5)	
Stress awareness			< 0.0001		< 0.0001			< 0.0001		< 0.0001
High	10,285 (30.9)	2266 (9.5)		451 (1.9)		13,673 (42.4)	3490 (14.6)		756 (3.2)	
Low	22,969 (69.1)	976 (2.4)		177 (0.4)		18,558 (57.6)	1036 (2.5)		216 (0.5)	
Depression			< 0.0001		< 0.0001			< 0.0001		< 0.0001
Yes	6655 (20.0)	2269 (14.5)		470 (3.0)		9043 (28.1)	3364 (21.4)		757 (4.8)	
No	26,599 (80.0)	973 (2.0)		158 (0.3)		23,188 (71.9)	1162 (2.3)		215 (0.4)	
Sleep time for fatigue recovery			< 0.0001		< 0.0001			< 0.0001		< 0.0001
Sufficient	10,762 (32.4)	627 (3.5)		126 (0.7)		7164 (22.2)	639 (3.6)		152 (0.8)	
Normal	11,413 (34.3)	995 (4.5)		178 (0.8)		10,830 (33.6)	1283 (5.8)		273 (1.2)	
Not sufficient	11,079 (33.3)	1620 (6.4)		324 (1.3)		14,237 (44.2)	2604 (10.3)		547 (2.2)	
Causes of stress			< 0.0001		< 0.0001			< 0.0001		< 0.0001
Achievement/career choice	19,123 (57.5)	1421 (3.7)		202 (0.5)		19,576 (60.7)	2042 (5.3)		347 (0.9)	
Family problems	6126 (18.4)	982 (9.4)		214 (2.0)		4360 (13.5)	1103 (10.5)		274 (2.6)	
Friendships	2539 (7.6)	386 (6.6)		79 (1.4)		3308 (10.3)	740 (12.7)		182 (3.1)	
Appearance	3160 (9.5)	231 (3.2)		57 (0.8)		4002 (12.4)	496 (6.9)		125 (1.7)	
Other	2306 (6.9)	222 (6.7)		76 (2.3)		985 (3.1)	145 (4.4)		44 (1.3)	
Total	33,254	3242 (9.7)		628 (1.9)		32,231	4526 (14.0)		972 (3.0)	

Among the participants with depression, suicidal ideation was reported in 14.5% (n = 2269) of male and 21.4% (n = 3364) of female participants, and suicide attempts were reported by 3.0% (n = 470) of male participants and 4.8% (n = 757) of female participants. Regarding suicide attempts by male participants, all P values for the proposed variables revealed statistical significance, except for those for middle and high school. Stress was caused by achievement/career choice in 57.5% (n = 19,123) of male participants and 60.7% (n = 19,576) of female participants, family problems in 18.4% of male and 13.5% of female participants, and by appearance in 9.5% of male and 12.4% of female participants.

The ORs of factors associated with suicidal ideation/suicide attempts are presented in Table 2 according to sex. Male participants who were counseled by their mother (OR 0.80; 95% CI 0.70–0.90) or a friend (OR 0.89; 95% CI 0.80–0.99) were less likely to think about suicide compared to those who had no counselor; those who were counseled by a friend (OR 0.74; 95% CI 0.60–0.92) were less likely to attempt suicide compared to those who had no counselor. Regarding suicidal ideation in female participants, the ORs of suicidal ideation in all types of counselors were statistically low, except for fathers and teachers. The OR of suicide attempts in female participants who had been counseled by their mother and friend was 0.66-fold higher (95% CI 0.53–0.82) and 0.70-fold higher (95% CI 0.58–0.83), respectively, than those of female participants with no counselor. In particular, male and female participants who were depressed had an 8.15 and 6.85 times greater risk of suicidal ideation and a 7.08 and 5.32 times greater risk for attempting suicide, respectively, compared to those without depression. With regard to the causes of stress, male participants were more likely to think about suicide due to problems with their family (OR 1.58; 95% CI 1.42–1.75) or friends (OR 1.29; 95% CI 1.12–1.48) than due to achievement/career choice issues. Similarly, female participants were more likely to consider suicide due to problems with their family (OR 1.62; 95% CI 1.47–1.80) or friends (OR 1.30; 95% CI 1.16–1.45) than due to achievement/career choice issues. In terms of causes of stress, all ORs for suicide attempt were significantly higher compared to that of achievement/career choice for both sexes.

**Table 2:** Factors associated with suicidal ideation/suicide attempts

Variables	Male participants				Female participants			
	Suicidal ideation		Suicide attempt		Suicidal ideation		Suicide attempt	
	Adj. OR	95% CI	Adj. OR	95% CI	Adj. OR	95% CI	Adj. OR	95% CI
Type of counselor								
Father	1.03	(0.86–1.23)	1.15	(0.83–1.61)	0.82	(0.63–1.08)	1.20	(0.79–1.84)
Mother	0.80	(0.70–0.90)	0.83	(0.64–1.07)	0.61	(0.54–0.68)	0.66	(0.53–0.82)
Sibling	1.01	(0.81–1.26)	1.10	(0.74–1.65)	0.61	(0.51–0.72)	0.74	(0.55–1.01)
Friend	0.89	(0.80–0.99)	0.74	(0.60–0.92)	0.69	(0.62–0.76)	0.70	(0.58–0.83)
Teacher	1.16	(0.91–1.47)	1.18	(0.77–1.82)	0.82	(0.61–1.11)	0.95	(0.60–1.51)
Other	1.25	(0.99–1.57)	1.37	(0.92–2.04)	0.66	(0.52–0.83)	0.91	(0.62–1.34)
None	1.00		1.00		1.00		1.00	
Middle and high school								
Middle school	1.30	(1.18–1.42)	1.68	(1.40–2.02)	1.47	(1.36–1.60)	2.06	(1.40–2.06)
High school	1.00		1.00		1.00		1.00	
Having parents								
Both or single parents	0.52	(0.40–0.68)	0.34	(0.24–0.49)	0.90	(0.63–1.31)	0.44	(0.23–0.50)
None	1.00		1.00		1.00		1.00	
Perceived household economic status								
High	1.00	(0.89–1.13)	1.01	(0.81–1.26)	0.85	(0.77–0.95)	0.97	(0.84–1.33)
Middle	0.88	(0.79–0.99)	0.81	(0.65–1.00)	0.74	(0.67–0.82)	0.71	(0.67–1.05)
Low	1.00		1.00		1.00		1.00	
Academic achievement								
High	1.04	(0.94–1.15)	0.93	(0.76–1.13)	1.12	(1.02–1.23)	0.91	(0.71–1.07)
Middle	1.04	(0.93–1.16)	0.96	(0.78–1.19)	0.99	(0.90–1.09)	0.94	(0.76–1.17)
Low	1.00		1.00		1.00		1.00	
Alcohol								
Yes	1.20	(1.07–1.34)	1.23	(0.99–1.53)	1.24	(1.11–1.38)	1.38	(0.95–1.47)
No	1.00		1.00		1.00		1.00	
Smoking								
Yes	1.06	(0.93–1.21)	1.87	(1.49–2.35)	1.41	(1.18–1.68)	2.30	(1.49–2.37)
No	1.00		1.00		1.00		1.00	
Subjective happiness								
Happy	1.00				1.00		1.00	
Neutral	1.50	(1.36–1.66)	1.24	(1.01–1.53)	1.51	(1.38–1.64)	1.25	(1.05–1.49)
Unhappy	3.32	(2.94–3.75)	1.92	(1.52–2.41)	3.46	(3.09–3.88)	2.70	(2.23–3.28)
Stress awareness								
High	2.43	(2.21–2.67)	2.20	(1.79–2.69)	2.34	(2.15–2.55)	1.91	(1.60–2.28)
Low	1.00		1.00		1.00		1.00	
Depression								
Yes	8.15	(7.46–8.90)	7.08	(5.81–8.63)	6.85	(6.33–7.40)	5.32	(4.51–6.28)
No	1.00		1.00		1.00		1.00	
Sleep time for fatigue recovery								
Sufficient	0.78	(0.70–0.88)	0.79	(0.63–0.99)	0.85	(0.76–0.95)	0.98	(0.80–1.20)
Normal	0.92	(0.83–1.01)	0.83	(0.68–1.01)	0.85	(0.78–0.93)	0.90	(0.77–1.06)
Not sufficient	1.00		1.00		1.00		1.00	
Causes of stress								
Achievement/career choice	1.00		1.00		1.00		1.00	
Family problems	1.58	(1.42–1.75)	1.91	(1.55–2.36)	1.62	(1.47–1.80)	1.68	(1.41–2.01)
Friendships	1.29	(1.12–1.48)	1.64	(1.25–2.16)	1.30	(1.16–1.45)	1.49	(1.23–1.82)
Appearance	0.95	(0.80–1.11)	1.53	(1.12–2.08)	1.01	(0.90–1.14)	1.30	(1.05–1.62)
Other	1.10	(0.93–1.31)	2.42	(1.83–3.21)	1.03	(0.83–1.27)	1.51	(1.07–2.13)

*CI* confidence interval, *OR* odds ratio

Table 3 shows a subgroup analysis of the associations between the types of counselor and suicidal ideation/suicide attempt stratified by causes of stress for both sexes. Male participants who were counseled by their mothers were less likely to think about suicide when they had achievement/career choice (OR 0.75; 95% CI 0.63–0.89) or family (OR 0.74; 95% CI 0.57–0.97) problems than participants with no counselor, and those who were counseled by a friend were less likely to attempt suicide when they were experiencing achievement/career choice (OR 0.66; 95% CI 0.45–0.96) or appearance (OR 0.50; 95% CI 0.25–0.99) problems compared to participants without counselor. On the other hand, female participants who were counseled by anyone except their father were less likely to think about suicide when they had achievement/career problems than participants without counselor, and those who were counseled by their mother or friend were less likely to attempt suicide when they were experiencing achievement/career choice or friend problems compared to those without any counselor.

**Table 3:** Subgroup analysis of associations between types of counselor and suicidal ideation/suicide attempt stratified by cause of stress

	None	Father		Mother			Brother, sister		Friend		Teacher		Others	
	Adj. OR	Adj. OR	95% CI	Adj. OR		95% CI	Adj. OR	95% CI	Adj. OR	95% CI	Adj. OR	95% CI	Adj. OR	95% CI
Male participants														
Suicidal ideation														
Causes of stress														
Achievement/career choice	1.00	0.94	(0.72–1.22)	0.75	(0.63–0.89)		1.01	(0.74–1.39)	0.89	(0.76–1.05)	0.90	(0.61–1.33)	1.12	(0.78–1.60)
Family problem	1.00	1.25	(0.89–1.76)	0.74	(0.57–0.97)		1.00	(0.65–1.54)	0.96	(0.79–1.17)	1.13	(0.73–1.74)	1.08	(0.71–1.64)
Friendships	1.00	0.76	(0.43–1.34)	0.97	(0.66–1.42)		1.19	(0.68–2.09)	0.82	(0.59–1.14)	1.47	(0.81–2.65)	1.91	(0.98–3.71)
Appearance	1.00	1.71	(0.94–3.12)	0.82	(0.50–1.34)		0.74	(0.30–1.86)	0.95	(0.64–1.41)	3.17	(1.45–6.94)	1.97	(0.92–4.21)
Other	1.00	0.76	(0.39–1.49)	1.03	(0.64–1.64)		0.83	(0.35–1.94)	0.71	(0.45–1.11)	0.81	(0.25–2.64)	1.17	(0.52–2.63)
Suicide attempt														
Causes of stress														
Achievement/career choice	1.00	1.15	(0.66–1.99)	0.69	(0.45–1.05)		0.92	(0.44–1.90)	0.66	(0.45–0.96)	0.60	(0.21–1.69)	1.41	(0.70–2.84)
Family problem	1.00	1.85	(1.05–3.28)	1.12	(0.70–1.81)		1.46	(0.72–2.97)	0.90	(0.62–1.30)	1.48	(0.73–3.02)	1.60	(0.83–3.10)
Friendships	1.00	0.95	(0.34–2.64)	1.11	(0.55–2.24)		0.93	(0.30–2.85)	0.75	(0.40–1.42)	2.24	(0.89–5.68)	0.76	(0.17–3.50)
Appearance	1.00	0.59	(0.17–2.09)	0.37	(0.13–1.01)		1.07	(0.32–3.50)	0.50	(0.25–0.99)	0.90	(0.23–3.54)	0.65	(0.14–3.04)
Other	1.00	0.65	(0.23–1.86)	0.78	(0.37–1.66)		0.74	(0.22–2.50)	0.84	(0.43–1.61)	1.16	(0.23–5.79)	1.57	(0.55–4.43)
Female participants														
Suicidal ideation														
Causes of stress														
Achievement/career choice	1.00	0.72	(0.48–1.07)	0.54	(0.46–0.64)		0.55	(0.43–0.70)	0.65	(0.56–0.75)	0.57	(0.34–0.97)	0.58	(0.40–0.83)
Family problem	1.00	0.44	(0.22–0.88)	0.58	(0.43–0.77)		0.85	(0.58–1.24)	0.72	(0.59–0.88)	0.97	(0.56–1.70)	0.92	(0.59–1.42)
Friendships	1.00	1.09	(0.56–2.13)	0.79	(0.58–1.07)		0.53	(0.33–0.84)	0.76	(0.58–1.01)	1.08	(0.57–2.08)	0.57	(0.30–1.06)
Appearance	1.00	1.30	(0.63–2.70)	0.76	(0.54–1.08)		0.63	(0.38–1.04)	0.69	(0.51–0.93)	1.05	(0.47–2.38)	0.61	(0.31–1.21)
Other	1.00	1.56	(0.53–4.53)	0.56	(0.30–1.06)		0.84	(0.32–2.21)	0.63	(0.35–1.13)	0.57	(0.05–6.09)	0.54	(0.15–1.95)
Suicide attempt														
Causes of stress														
Achievement, Career choice	1.00	1.02	(0.48–2.18)	0.65	(0.46–0.91)		0.74	(0.45–1.22)	0.73	(0.55–0.98)	0.38	(0.09–1.59)	0.82	(0.40–1.69)
Family problem	1.00	0.58	(0.19–1.73)	0.54	(0.32–0.92)		0.75	(0.40–1.40)	0.77	(0.57–1.05)	0.95	(0.42–2.15)	1.01	(0.53–1.93)
Friendships	1.00	1.57	(0.62–3.98)	0.69	(0.42–1.11)		0.51	(0.23–1.16)	0.58	(0.38–0.89)	1.51	(0.68–3.32)	0.71	(0.26–1.91)
Appearance	1.00	2.00	(0.69–5.83)	1.06	(0.58–1.94)		1.07	(0.47–2.45)	0.77	(0.45–1.30)	1.03	(0.27–3.92)	1.61	(0.63–4.12)
Other	1.00	1.60	(0.36–7.03)	0.36	(0.13–1.03)		1.00	(0.26–3.91)	0.41	(0.16–1.05)	0.71	(0.04–12.41)	0.47	(0.05–4.32)

The odds ratios are adjusted for covariates

*CI* confidence interval, *OR* odds ratio

## Discussion

In the present study, we used nationally represented data to examine whether having a counselor was significantly associated with suicidal ideation/suicide attempts in adolescents and whether counselor type had an effect. We found that each type of counselor had a different association with suicidal ideation/suicide attempts in both male and female participants. In addition, associations between the type of counselor and suicidal ideation/suicide attempts were significantly influenced by level of education, having parents, subjective happiness, stress awareness, depression, and causes of stress.

We found that female participants tended to have more suicidal thoughts and attempts than males did, which supports previous findings [21–24]. Male participants were more likely than female participants to have no counselors; female participants were mostly counseled by friends. As women tend to actively communicate with those around them, they are perhaps more likely than men to have a counselor; conversely, men in Korea may not be familiar with expressing their difficulties to others, so they may be forced to suppress emotions (such as sadness) [25]. Teachers accounted for the lowest proportion of counselors for both sexes, which is suggestive of a general lack of communication with teachers.

Previous research has reported that psychological stability can be felt through intimate emotional ties with the family during adolescence [26]. Therefore, we hypothesized that finding counsel with one’s mother may be the most effective for reducing the risk of suicidal ideations/suicide attempts. However, we found that the likelihood of suicidal thoughts/suicide attempts was not always the lowest when the counselor was a mother.

The OR for suicidal ideation/suicide attempts was significantly low for both sexes when they were counseled by friends, which suggests that not only family members can prevent suicides but also friends can do the same. Social relationships significantly affect adolescents’ physical and mental health; thus, a good relationship with not only family members but also friends could lower the risk of suicidal ideation [27]. Female participants were significantly less likely to experience suicidal ideation when they confided in their family members (except for their father) and another person (including a friend) compared to participants who did not receive any counselling, which indicates that women were more easily psychologically affected by people around them than were men, for whom only counsel from mothers or friends seemed to have significant effects. A previous study showed that adolescent girls typically perceived higher levels of social support from their friends than did boys with similar levels of adult support [28]. However, social support is still important for mental health of both sexes [29]. Regarding suicide attempts, friends may be the most effective in reducing the risk of suicide attempts for male participants, whereas mothers and friends were the most effective for female participants. As adolescents spend most of their time in school, support from their friends might be highly associated with depression and suicidal ideation in both sexes [28].

There was a higher risk of suicide among middle school students than among high school students. We also observed an increased risk of suicide attempts among adolescents in no-parent families than among those with parents. A previous study also showed that adolescents with single-parent families suffer from mental health problems more frequently than do adolescents with two-parent families as the former spend less time with their parents (e.g., having a conversation) that do the latter [30]. Moreover, the risk of suicidal ideation in female participants increased when their academic performance was high. Although both boys and girls have the same levels of worry regarding academic performance, girls are more vulnerable to increased stress due to comparison with their classmates [31]. Moreover, one study showed that adolescent girls experience more stress than boys do [31, 32]. A previous study reported that worries about academic performance among adolescents induces insufficient sleep, as many adolescents believe that academic performance is important for preparing themselves for adulthood [19, 33]. One study showed that adolescents who usually go to bed late due to worries about academic performance in school may have an increased risk of suicidal ideation [19]. Sufficient sleep time in both sexes significantly decreased suicidal ideation compared to insufficient sleep time [19]. This suggests that adequate sleep time can be a good preventive measure against suicide among adolescents.

Overall, risk factors such as subjective happiness, stress awareness, and depression, which have been found to have a significant impact on suicide attempts in previous studies, had lower ORs in those who had attempted suicide than in those who had thought about suicide [34, 35]. This finding can be interpreted in the context of previous studies. Suicide attempts in adolescents tend to occur spontaneously and accidentally, rather than deliberately. This impulsiveness reflects an unstable psychological state rather than a series of continuous processes (think about suicide, make a detailed plan for it, and then attempt or enforce it) [36–38].

We identified differences in the types of counselors who were effective for preventing suicidal ideation/suicide attempts among male and female participants according to the causes of stress, which significantly reduced the risk of suicide. Adolescents most commonly experienced stress caused by academic performance or career choice anxiety, but the highest risk of suicidal thoughts and suicide attempts was associated with family stress. Stress levels are increased by the burden on the grades and choices of career rather than on itself, which increases the feeling of depression and further causes suicidal thoughts/suicide attempts [39]. In both male and female participants, appearance and other problems were significantly associated with suicide attempts, which suggests that suicide in adolescents is a very complex issue.

The subgroup analysis revealed that male participants were less likely to think about suicide when they were counseled by their mother about family problems compared to those without counselor; however, there was an increased risk of suicide by a factor of 1.85 when they were counseled by their father. A previous study suggested that fathers have difficulties in fulfilling a counselor’s role due to a lack of absolute communication time with their children [40]; thus, the risk of suicide in youths is likely to be high when there is a conflict with their fathers in terms of family problems. On the other hand, female participants with a family problem had a significantly reduced likelihood of suicidal thoughts when they were counseled by their fathers, which suggests that fathers can also successfully play a counselor’s role. A previous study showed that over half of the girls and one-third of boys felt that they could not be counseled by their fathers [41]. Therefore, the few adolescents who are counseled by their fathers probably have good relationships with their fathers, which may also be related to their low risk for suicidal ideation [41, 42]. Moreover, a previous study showed that depression and suicide are associated with the perceived quality of adolescents’ relationships with their parents [43]. Although most Korean adolescents know the necessity of counseling and wish to confide in someone, 88.25% do not want counseling at school because of the one-sided counseling methods used [44]. In addition, 40.4% of adolescents in a previous study reported that one-sided counseling was not helpful. However, female participants with achievement/career choice-related problems were less likely to think about suicide when they were counseled by their teacher than when they had no counselor. This suggests that the role of teachers as counselors in preventing suicide should be emphasized, especially considering that adolescents spend most of their time in school and face many academic and career choice problems.

The present study had several limitations. First, as a result of this study’s cross-sectional design, cause and effect, as well as the direction of the relationships observed, could not be determined. Second, this study was based on anonymous online surveys, and adolescents may have under- or over-reported characteristics. Therefore, responses could have been affected by social desirability bias. There is a possibility that some adolescents may have expressed their feelings with a desire to let others know about their depression and suffering (stress), which could have affected the study results [45, 46]. Moreover, as there is a tendency for respondents to show reluctance to answer questions related to suicide, there might have been more people in the sample with suicidal ideation and suicide planning than in the general population. Third, limited information was obtained through the questionnaires. Therefore, it was not possible to examine the interactions between the respondents and their counselors (such as counseling frequency and quality) in more detail. Fourth, the only available results regarding causes of stress were from 2015, and the KYRBWS data for other years could not be obtained. Lastly, due to lack of data, suicidal ideation, suicide attempts, and depression were measured using responses to self-reported dichotomous questions. Therefore, further research using segmented measurements accompanied by experts is needed.

Despite these limitations, the present study has several strengths. To our knowledge, this study is the first to investigate the association between type of counselors and suicidal ideation/suicide attempts. Logistic regression was used to confirm the unique characteristics of South Korean adolescents and their association with suicidal ideation/suicide attempts. Furthermore, as this study analyzed data from the KYRBWS, the results can be generalized to the whole population of South Korean adolescents. The results of this study could be helpful for youth mental health promotion and psychological counseling research or be used as basic data for suicide prevention policy development. In addition, the data in this study can be used by youth counseling welfare centers, youth companions (a counseling specialist), or other youth protection services.

## Conclusions

The current study identified a significant relationship between the type of counselor, suicidal ideation, and suicide attempts. Our findings suggest that adolescents who were counseled by family, friends, teachers, or others were less likely to show suicidal ideation and suicide planning than were those who did not receive counseling. Although there was a difference between sexes in relation to the type of counselor that was effective, we found that receiving counseling was beneficial to the mental health of both male and female adolescents. Therefore, our results seem to suggest that it is necessary to lower the communication barriers of adolescents and listen carefully to their worries. As 60% of individuals transition from suicidal ideation to suicide planning and attempts occur within the first year of the onset of ideation, it is important to prevent suicidal ideation and suicide planning [19]. Furthermore, as having a counselor had a significant influence on suicidal ideation/suicide attempts among adolescents, it is necessary to create an environment in which adolescents can confide in the people around them. Although almost everyone could be a counselor for troubled youths, the effectiveness of each counselor type may vary according to sex. Finally, adults should pay close attention to adolescents, and adolescents need to learn how to form mutually supportive relationships through active communication with those around them.

## Data Availability

The datasets were accessed via https://www.kdca.go.kr/yhs/home.jsp with the approval of the Korea Centers for Disease Control and Prevention (KCDC) after submission of a written oath and data utilization plan.

## Abbreviations

OECD: Organization for Economic Co-operation and Development
KYRBWS: Korea Youth Risk Behavior Web-based Survey
KCDC: Korea Centers for Disease Control and Prevention
OR: Odds ratio
CI: Confidence interval
